# 54101 Characterizing Microbiota Features of Clostridioides difficile Infections

**DOI:** 10.1017/cts.2021.631

**Published:** 2021-03-31

**Authors:** Sarah Tomkovich, Krishna Rao, Vincent B. Young, Patrick D. Schloss

**Affiliations:** University of Michigan

## Abstract

ABSTRACT IMPACT: Our goal is to identify bacterial biomarkers of adverse Clostridioides difficile infection outcomes OBJECTIVES/GOALS: We characterized microbiota features of Clostridioides difficile infections (CDIs) and will investigate the association between bacterial taxa and adverse outcomes, which includes severe and recurrent CDIs. METHODS/STUDY POPULATION: 1,517 stool samples were collected from patients diagnosed with a CDI at the University of Michigan along with 1,516 unformed and 910 formed stool control samples. We characterized the microbiota of the 3,943 stool samples by sequencing the V4 region of the 16S rRNA gene and used the Dirichlet Multinomial Mixtures method to cluster samples into community types. Severe CDI cases were defined using the Infectious Diseases Society of America criteria and recurrent CDIs were defined as CDIs that occurred within 2-12 weeks of the primary CDI. We will use machine learning to examine whether specific bacterial taxa can predict severe or recurrent CDIs. We will test 5 machine learning models with 80% training and 20% testing data split. RESULTS/ANTICIPATED RESULTS: Similar to findings from a previous study with 338 samples, we found there was no difference in diversity between CDI cases and unformed controls (Inverse Simpson index, p > 0.5) and samples from the 3 groups (CDIs, unformed controls, and formed controls) clustered into 12 community types. To investigate the bacterial taxa that are important for predicting adverse CDI outcomes, we will select the best machine learning model based on performance and training time and examine how much each feature contributes to performance. We anticipate the large number of CDI cases in our cohort and robust machine learning approaches will enable us to identify more bacteria associated with adverse outcomes compared to other studies that have attempted to predict CDI recurrence with fewer CDI cases. DISCUSSION/SIGNIFICANCE OF FINDINGS: Adverse CDI outcomes are a significant source of the morbidities, mortalities, and healthcare costs associated with CDIs. Identifying bacterial biomarkers of severe and recurrent CDIs could enhance our ability to stratify patients into risk groups and may lead to the development of more targeted therapeutics.

